# Ultra-Sensitive Analysis of Organophosphorus Compounds by Comparative GC-FPD and GC-ICP-MS: Implications for Chemical Warfare Agent Detection

**DOI:** 10.3390/molecules30204086

**Published:** 2025-10-14

**Authors:** Michał Wiktorko, Piotr Kot, Anna Puchała, Patrycja Bryczek-Wróbel, Klaudia Izabela Rzadkowska, Barbara Wiaderek

**Affiliations:** 1 Military Institute of Chemistry and Radiometry, ul. Gen. A. Chruściela “Montera” 105, 00-910 Warsaw, Poland; 2 Faculty of Security, Logistics and Management, Military University of Technology, ul. Gen. S. Kaliskiego 2, 00-908 Warsaw, Poland

**Keywords:** chemical safety, gas chromatography (GC-FPD), GC-ICP-MS, organophosphorus nerve agents (GB, GD, GF)

## Abstract

Organophosphorus chemical warfare agents such as sarin (GB), soman (GD), and cyclosarin (GF) rank among the most toxic substances known, making trace-level detection critical for public and military safety. In this study, we compared the sensitivity of two analytical techniques for determining these nerve agents: gas chromatography with flame-photometric detection (GC-FPD) and gas chromatography coupled to inductively coupled plasma mass spectrometry (GC-ICP-MS). Diluted samples of sarin, soman, and cyclosarin were prepared under controlled laboratory conditions and then analyzed by both methods. Limits of detection, calibration linearity, and selectivity of the two approaches were evaluated. It was shown that GC-ICP-MS enabled detection of sarin, soman, and cyclosarin at ≈0.12–0.14 ng/mL (LOD), whereas GC-FPD achieved LODs of ≈0.36–0.43 ng/mL. The obtained results confirm that GC-ICP-MS exhibits significantly higher sensitivity than GC-FPD in the analysis of the chemical warfare agents under study. This advantage indicates strong application potential of GC-ICP-MS as a technique for ultra-sensitive detection of trace amounts of chemical warfare agents (CWAs) in environmental samples and in confirmatory testing for compliance with the CWC, while simultaneously employing GC-FPD for rapid preliminary monitoring.

## 1. Introduction

**Figure 1:**
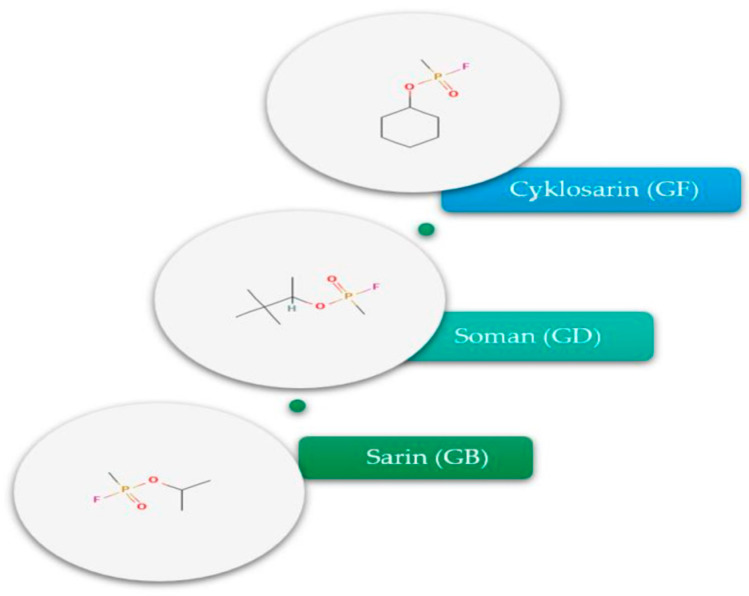
Structural formulas G-series [2].

Sarin (isopropyl methylphosphonofluoridate), soman (pinacolyl methylphosphonofluoridate), and cyclosarin (cyclohexyl methylphosphonofluoridate) (Figure 1) are classical nerve chemical warfare agents classified as organophosphorus acetylcholinesterase inhibitors. They are characterized by extremely high toxicity—even concentrations in the ppm range and below pose a lethal hazard to humans (the LCt_50_ for sarin is estimated at ≈70 µg·min/L). Despite the passage of several decades since their invention and the introduction of a ban on their use, these agents continue to pose a real threat, as demonstrated by terrorist attacks and battlefield use—among others, the 1995 Tokyo subway sarin attack and the confirmed use of sarin during the Syrian conflict in 2013–2017 [1,2,3]. Therefore, there is a need for reliable detection methods for these compounds in environmental and forensic samples, particularly when these are present in trace amounts.

Classical analytical techniques used for the detection and identification of organophosphorus chemical warfare agents include chromatographic methods, particularly gas chromatography coupled with selective detectors and/or mass spectrometers. Gas chromatography with flame photometric detection (GC-FPD) has long been employed for determining compounds containing phosphorus and sulfur—including pesticides and chemical warfare agents—owing to the FPD detector’s selectivity toward P/S heteroatoms [4]. The FPD records element-specific emission generated in a hydrogen–air flame; using a band-pass filter that transmits the P (~526 nm) or S (~394 nm) emission lines enables selective detection [5]. Typical FPD detection limits for organophosphorus compounds are on the order of ~10 ng/mL for a 1.0 µL injection. The pulsed variant (PFPD) offers sub-picogram detectivity (manufacturer specification ≤0.1 pg P·s^−1^, in newer designs ≤100 fg P·s^−1^); for consistency, practical method LODs are reported here as on-column mass (pg) and the equivalent solution concentration (ng/mL) [6,7,8,9]. Despite this sensitivity, GC-FPD is often insufficient for detecting ultra-low concentrations of these CWAs in complex environmental matrices. Moreover, the flame detector can suffer from signal quenching in the presence of large amounts of co-eluting substances, which limits its usefulness for the analysis of real environmental samples with complex composition.

The development of detection methods based on inductively coupled plasma mass spectrometry (ICP-MS) has opened new possibilities for ultra-sensitive analysis of compounds containing heteroatoms such as phosphorus, sulfur, or arsenic. ICP-MS, particularly when coupled with chromatography, offers exceptionally low detection limits reaching the parts-per-trillion (ppt) level and a wide dynamic range of quantification [1]. The detection principle involves dissociating analyte molecules in an argon plasma into atoms and measuring their masses (e.g., ^31^P^+^ for phosphorus) using a mass spectrometer. An advantage of this approach is elemental selectivity-the ICP-MS detector records signal exclusively from the selected isotope (e.g., ^31^P), which eliminates many potential interferences arising from inorganic compounds or other matrix components [10]. In cases where the degradation products of G-agents, such as alkyl methylphosphonic acids, are subject to analysis, the situation is different—these compounds are polar, strongly hydrophilic, and essentially non-volatile under gas chromatographic conditions. Without prior chemical modification, they cannot be introduced into or effectively separated on a GC column. Therefore, derivatization is employed prior to their analysis, most commonly through silylation (e.g., to TBDMS derivatives), which imparts a more hydrophobic and volatile character, thereby enabling transport in the gas phase, effective chromatographic separation, and subsequent detection by ICP-MS [11]. Early demonstration studies have shown that GC-ICP-MS can detect derivatized phosphonic acids with a detection limit of <5 pg on-column [1], representing a two-order-of-magnitude improvement relative to GC-FPD. Moreover, introducing the sample in the gas phase into the plasma (as opposed to conventional HPLC-ICP-MS from solution) results in a lower background for the ^31^P signal due to reduced presence of interfering nitrogen oxide ions from air [1].

The aim of this study is a comparative assessment of the sensitivity of GC-FPD and GC-ICP-MS in the analysis of trace amounts of sarin, soman, and cyclosarin. Detection limits (LOD) and limits of quantification (LOQ) were determined and compared for both techniques, repeatability and response linearity were evaluated, and the practical potential of both methods for the detection of chemical warfare agents was analyzed. Particular attention was paid to the applicability of the methods for identifying trace amounts of CWAs-both in rapid field monitoring (GC-FPD as a portable detector) and for laboratory confirmation at the highest level of analytical confidence (GC-ICP-MS as a confirmatory method).

## 2. Results and Discussion

The results presented in Table 1 confirm that both techniques-GC-FPD and GC-ICP-MS-exhibit excellent detector response linearity over a wide concentration range (1 ng/mL to 10 µg/mL) for all G-series compounds examined. Coefficients of determination (R^2^) on the order of 0.9999–0.999999 indicate an outstanding correlation between detector signal and analyte concentration, fully consistent with prior reports employing gas chromatography for the analysis of organophosphorus nerve agents (OPNAs) [1,12]. Both methods also demonstrated very high analytical sensitivity. The limits of detection (LOD) and quantification (LOQ) for both methods (GC-ICP-MS and GC-FPD) were determined based on a minimum of seven independent replicate analyses for each compound, ensuring appropriate repeatability and statistical reliability of the results. The limits of detection (LOD) obtained in this work reach ~0.1 ng/mL, and the limits of quantification (LOQ) fall within 0.1–1 ng/mL. Differences in sensitivity between GC-FPD and GC-ICP-MS are slight (on the order of 0.02–0.03 ng/mL in favor of GC-ICP-MS), yet they corroborate the advantage of the ICP-MS detector for ultra-trace detection. Particularly important here is the elemental selectivity of ICP-MS for the phosphorus isotope (^31^P), which translates into an ability to capture trace amounts of organophosphorus compounds in the presence of complex matrices. The practical relevance of achieving such ultra-trace detection thresholds is underscored by recent work on barrier materials designed to limit exposure to toxic gases, where analytical capability at very low concentrations informs material performance assessment and risk reduction strategies [13]. The LOD/LOQ levels obtained are consistent with values reported in the primary literature. For example, picogram-level detection of nerve agents and their silylated breakdown products by GC-MS/MS has been demonstrated in experimental studies, while GC-ICP-MS(/MS) affords ultra-trace, element-selective detection with sub-ng/mL limits for phosphorus; recent OPNA-focused work also reports achievable LODs near 0.1 ng/mL for derivatized phosphonic acids. This is in line with the trends summarized by Valdez and Leif (review) and with the experimental results reported by Kuligowska and Neffe for ICP-MS-based approaches [1,11,14]. Similar sensitivity parameters are reported by the US EPA [15] for chromatographic determination of organophosphorus compounds, confirming the competitiveness of both methods investigated. It is worth emphasizing that although the LOD/LOQ differences between GC-FPD and GC-ICP-MS are small, GC-ICP-MS affords greater confidence in the ultra-trace region, which is critical in evidentiary analysis and environmental monitoring. In terms of precision, both techniques meet stringent analytical requirements. Repeatability (expressed as relative standard deviation, RSD) was better than 5% for all compounds and in both methods, indicating high stability and reliability of measurements. Such precision is essential for practical applications—both in OPCW reference laboratories and in military CBRN units—where each analysis must be reproducible and trustworthy. Our results are consistent with the literature, in which a comparable level of repeatability (<5% RSD) has been obtained for organophosphorus compounds using mass-spectrometric detectors (GC-MS, GC-MS/MS) [16]. From a practical standpoint, the results suggest complementary roles for the two techniques in the analysis of chemical warfare agents. GC-FPD emerges as an excellent screening technique—relatively simple to operate, less costly, and fast. It is therefore well suited to routine environmental monitoring (e.g., assessing contamination of soil, water, or air in post-attack zones) and to field operations. Portable gas chromatographs equipped with FPD can be used for rapid on-site detection of nerve agents. By contrast, GC-ICP-MS excels as a confirmatory method. Its unique sensitivity and elemental selectivity (e.g., specific detection of phosphorus) enable identification at ultra-low levels, which is often crucial in forensic and judicial investigations. From a safety and response perspective, studies on the thermal and calorimetric properties of mitigation agents (e.g., fire-extinguishing powders) highlight the need for robust analytical evidence at very low levels to support post-incident assessment and operational decision-making, further justifying the emphasis on ultra-trace capability reported here [17]. For example, this technique can confirm the presence of trace residues of sarin or soman in complex biological or environmental samples, providing strong evidentiary support. In the context of verifying international obligations (e.g., compliance with the Chemical Weapons Convention), GC-ICP-MS provides additional confidence and credibility of results thanks to selective detection of elements characteristic of chemical warfare agents.

## 3. Materials and Methods

### 3.1. Apparatus

An Agilent GC-ICP-MS system (Agilent Technologies, Shanghai, China) and an Agilent GC equipped with dual FPDs were used to compare the quantitative performance and sensitivity for the nerve agents sarin (GB), soman (GD), and cyclosarin (GF).

An Agilent 8890 gas chromatograph (Agilent Technologies, Shanghai, China) was fitted with an Rtx-OPPesticides capillary column (Restek, Centre County, PA, USA). The GC was coupled to an Agilent 7900 ICP-MS (Agilent Technologies) via the GC-ICP-MS interface (Agilent Technologies) using a heated transfer line. Two auxiliary heaters, controlled by the GC, maintained the transfer-line temperature. The column outlet was routed directly from the GC oven into the ICP torch to avoid condensation at the column–capillary junction. Instrument control and data acquisition were performed with Agilent ICP-MS MassHunter 5.3 Workstation software. Operating conditions for GC-ICP-MS are listed in Table 2.

A second Agilent 8890 GC (Agilent Technologies), configured with dual FPDs and the same Rtx-OPPesticides column (Restek, Centre County, PA, USA), was used for FPD measurements. Instrument control and data acquisition were performed with Agilent OpenLab ChemStation LTS 01.11 software. The operating conditions for GC-dual FPD are listed in Table 3.

### 3.2. Reagents

Sarin (GB, 98%), soman (GD, 99%), and cyclosarin (GF, 98%) were synthesized at the Military Institute of Chemistry and Radiometry. Hexane (GC, capillary grade; VWR Chemicals, Gdańsk, Poland) was used as the solvent.

### 3.3. Sample Preparation

Sarin (GB), soman (GD), and cyclosarin (GF) were diluted separately in hexane. Stock solutions (10 mg·cm^−3^; equivalent to 10 mg·mL^−1^) were prepared gravimetrically, and working solutions were obtained by serial dilution. Solution concentrations are listed in Table 4.

### 3.4. Mechanistic Basis for Performance Differences in GC-ICP-MS vs. GC-FPD

When analytes elute from the gas chromatograph into an inductively coupled plasma mass spectrometer (ICP-MS), they are entrained in an argon plasma at temperatures approaching 9000 K. In this environment, the aerosol is fully desolvated, and the molecules are atomized and ionized to elemental ions. The ion beam is then introduced into a mass analyzer and monitored at the isotope of interest; for organophosphorus compounds, this is ^31^P. Modern collision/reaction cell (CRC) designs use oxygen to convert ^31^P^+^ to its oxide (PO^+^) and thus shift detection from *m/z* 31 to *m/z* 47, or more generally to oxidize P and S to PO^+^ and SO^+^, effectively eliminating polyatomic interferences such as N_2_O^+^ and ^14^N^16^O^1^H^+^. These features explain why GC-ICP-MS provides element-selective, low-interference detection for phosphorus at ultra-trace levels [10]. In case of photometric detector FPD-the flame operates by burning analytes in a hydrogen–air flame and measuring element-specific chemiluminescence. Phosphorus-containing compounds are detected by their emission at approximately 526 nm, whereas sulfur compounds emit at 394 nm. The detector’s selectivity arises because only excited P- or S-containing fragments emit at these wavelengths. However, emission intensity is sensitive to the flame environment; large quantities of co-eluting hydrocarbons or other compounds consume oxygen or otherwise alter the flame composition, leading to quenching of the chemiluminescent signal. Application notes warn that such quenching appears as reduced and fluctuating responses in chromatograms and recommend optimizing the hydrogen–air ratio to mitigate this effect Consequently, although GC-FPD is highly sensitive for P and S, its signal can be suppressed in complex matrices [9].

## 4. Conclusions

The results obtained for sarin (GB), soman (GD), and cyclosarin (GF) using both methods demonstrated full complementarity. GC-FPD provided rapid, selective detection of these compounds at the ppm/ppb level, whereas GC-ICP-MS extended the detection range to the ultra-trace level, i.e., below the threshold attainable with a classical flame detector. This combination of analytical techniques constitutes an optimal approach to CWA analysis, coupling the speed and simplicity of screening with the ultra-high sensitivity and identification confidence afforded by confirmatory analysis. Importantly, the strategy of employing two independent detectors to verify concordant results aligns with OPCW recommendations to enhance analytical reliability through dual verification. It can therefore be concluded that the GC-FPD (screening) + GC-ICP-MS (confirmation) workflow significantly streamlines both monitoring activities (e.g., in environmental protection and public safety) and investigative procedures in suspected cases of prohibited chemical-agent use. Thus, the proposed set of methods provides robust, sensitive analytical tools for the scientific community and agencies responsible for public safety.

## Figures and Tables

**Table 1:** Comparison of validation parameters of the GC-FPD and GC-ICP-MS methods for sarin (GB), soman (GD), and cyclosarin (GF).

Compound (CWA)	Parameter	GC-FPD	GC-ICP-MS	Sensitivity Difference (ICP-MS vs. FPD)
Sarin (GB)	Linearity range [µg/mL]	0.001–10	0.001–10	–
	R^2^	0.9999	0.99999	–
	LOD [ng/mL]	120	10	≈12× lower
	LOQ [ng/mL]	360	30	≈12× lower
	Repeatability (%RSD)	<5%	<5%	comparable
Soman (GD)	Linearity range [µg/mL]	0.001–10	0.001–10	–
	R^2^	0.999999	0.99998	–
	LOD [ng/mL]	140	12	≈12× lower
	LOQ [ng/mL]	430	36	≈12× lower
	Repeatability (%RSD)	<5%	<5%	comparable
Cyclosarin (GF)	Linearity range [µg/mL]	0.001–10	0.001–10	–
	R^2^	0.9999	0.99997	–
	LOD [ng/mL]	130	11	≈12× lower
	LOQ [ng/mL]	390	33	≈12× lower
	Repeatability (%RSD)	<5%	<5%	comparable

**Table 2:** Operating conditions for GC-ICP-MS.

GC Column	Rtx-OPPesticides (30 m × 0.53 I.D. and 0.83 µm Film Thickness)
GC carrier gas	Nitrogen
GC carrier gas flow rate	5 mL/min
GC oven temperature	50 °C initial, hold 0.5 min, then ramped at 100 °C/min to 100 °C ramped at 80 °C/min to 100 °C ramped at 60 °C/min to 230 °C hold 0.2 min
GC injection volume	1 µL
GC injector temperature	200 °C
GC injection mode	Splitless
GC-ICP-MS transfer-line temperature	230 °C
ICP-MS Rf power	700 W
Dilution gas	Helium
Dilution gas flow rate	0.3 L/min
Dwell time	0.1 s per isotope
Isotopes monitored	^31^P

**Table 3:** Operating conditions for GC-dual FPD.

GC Column	Rtx-OPPesticides (30 m × 0.53 I.D. and 0.83 µm Film Thickness)
GC carrier gas	Nitrogen
GC carrier gas flow rate	5 mL/min
GC oven temperature	50 °C initial, hold 0.5 min, then ramped at 100 °C/min to 100 °C ramped at 80 °C/min to 100 °C ramped at 60 °C/min to 230 °C, hold 0.2 min
GC injection volume	1 µL
GC injector temperature	200 °C
GC injection mode	Splitless
Dual FPD temperature	200 °C
Dual FPD hydrogen flow rate	60 mL/min
Dual FPD air flow rate	92 mL/min
Dual FPD makeup flow rate	60 mL/min

**Table 4:** Solution concentrations.

Solution Number	Concentration [mg/cm^3^]
1	10
2	0.1
3	1 × 10^−2^
4	1 × 10^−3^
5	1 × 10^−4^
6	1 × 10^−5^
7	1 × 10^−6^

## Data Availability

Data are contained within the article.
